# Knee Osteoarthritis in Relation to the Risk Factors of the Metabolic Syndrome Components and Environment of Origin

**DOI:** 10.3390/jcm11247302

**Published:** 2022-12-08

**Authors:** Nicoleta Bianca Tudorachi, Tiberiu Totu, Iuliana Eva, Bogdan Bărbieru, Eugenia Eftimie Totu, Adrian Fifere, Tudor Pinteală, Paul-Dan Sîrbu, Valeriu Ardeleanu

**Affiliations:** 1 Faculty of Medicine, “Ovidius” University of Constanța, Mamaia Boulevard 124, 900527 Constanța, Romania; 2 Department of Health Sciences and Technology (D-HEST), ETH Zurich, 8093 Zurich, Switzerland; 3 Radiology and Medical Imaging Laboratory, “Iacob Czihac” Emergency Military Clinical Hospital, 7-9 General Henri Mathias Berthelot St., 700483 Iași, Romania; 4 Department of Orthopedics and Traumatology, “Iacob Czihac” Emergency Military Clinical Hospital, 7-9 General Henri Mathias Berthelot St., 700483 Iași, Romania; 5 Department of Analytical Chemistry and Environmental Engineering, Faculty of Industrial Chemistry and Biotechnologies, University Politehnica of Bucharest, 1-5 Polizu Street, Sector 1, 011061 Bucharest, Romania; 6 Centre of Advanced Research in Bionanoconjugates and Biopolymers, “Petru Poni” Institute of Macromolecular Chemistry, 41A Grigore Ghica Voda Alley, 700487 Iași, Romania; 7 Department of Orthopedics and Traumatology, Faculty of Medicine, Grigore T Popa University of Medicine and Pharmacy, 16 University Street, 7001 Iași, Romania

**Keywords:** knee osteoarthritis, metabolic syndrome, environment of origin, dyslipidemia, diabetes mellitus

## Abstract

Background: Knee osteoarthritis (KOA) is a chronic degenerative pathology that is associated with multiple risk factors such as age, sex, obesity, or metabolic syndrome (MetS). The present clinical trial aimed to investigate the influence of the environment of origin, body mass index (BMI), and MetS parameters on the KOA differentiated degrees. Methods: 85 patients were admitted for the clinical study. The KOA presence was investigated using X-rays analysis. The Kellgren–Lawrence classification (KL) of the KOA severity and the MetS characteristic parameters using freshly collected blood were performed for each patient. All data collected were used for ANOVA statistic interpretation. Results: The total cholesterol and glycemia were found to be statistically significant (*p* < 0.028, and *p* < 0.03, respectively), with a high level in patients with severe KOA compared to healthy ones. Patients from rural regions are 5.18 times more prone to develop severe KOA when compared to ones from urban areas. Conclusions: The results of the statistical analysis confirmed the correlation between the incidence and severity of KOA and the influence of increased values of BMI, glycemia, triglycerides, and total cholesterol. The investigations revealed a statistically significant influence of the environment of origin on the KOA degree of the patients.

## 1. Introduction

KOA is a chronic degenerative pathology representing one of the population’s leading causes of disability. Analyzing KOA evolution over time, it is easily observed that its prevalence has doubled since the middle of the 20th century [1]. In this sense, a recent meta-analysis highlights the global prevalence of KOA at a rate of 16% and an incidence of 203 per 10,000 persons/year [2]. The association between obesity, hyperglycemia, dyslipidemia, hypertension (MetS characteristic elements), and KOA is commonly highlighted nowadays. Risk factors such as age, gender, inflammation, or the presence of MetS [3,4], which are generally associated, can affect the knee joint over time [3], generating the onset and slow progression of KOA [5]. Certain sports, such as football or athletics, are also considered risk factors for KOA [6]; in professional football players, KOA can be regarded as a professional disease because the risk of presenting the pathology is two or three times higher than in the general population due to trauma [7]. Joint trauma is a significant risk factor that increases several times the risk of the development and progression of KOA; furthermore, it is well-documented that ligamentous or meniscal injuries are also associated with KOA [8]. In the case of other joints such as the hip, MetS does not show significant associations with arthritic changes, only elevated glycemia values [9]; although ankle osteoarthritis is less common, and in 80% of cases, it has a post-traumatic etiology [10].

The complex pathophysiology of KOA still requires research with respect to detecting and reducing modifiable risk factors and uncovering protective factors to help develop appropriate prevention strategies [11].

KOA is distinguished by three main symptoms: persistent knee pain, joint stiffness, and movement restriction, which may be accompanied by signs such as crepitus or swelling [12]. In many cases, KOA can progress silently so that when symptoms appear, imaging changes at the joint level are already detectable [5]. However, pain is the most common symptom that prompts patients to seek specialized medical attention. Furthermore, pain affects patients’ social and professional lives in advanced stages, eventually leading to disability [13]. KOA has an individualized evolution, and since it is multifactorial, it is considered a syndrome with several phenotypes [14]. The phenotype is represented by one or more characteristics of the pathology, with the help of which personalized treatment could be created to achieve better results than in the case of a standard generalized treatment [15]. So, in addition to the chronic pain [16] or inflammatory [17] phenotype, KOA was also included in the metabolic phenotype [18]. As a consequence of the suggested connection between KOA and the MetS, the metabolic KOA term was introduced [19].

MetS is characterized by a combination of factors such as obesity, hyperglycemia, dyslipidemia, and hypertension [20]. Its prevalence has increased with the general population lifestyle changes, being approximately 10–30% worldwide and 59% in patients with KOA. The association between KOA and MetS results in a more rapid onset of symptoms found to be more severe in these cases [19]. The explanation may lie in the existence of low levels of systemic inflammation at the knee joint level and the local accumulation of degradation products favored by the presence of obesity or hyperglycemia [4]. Diabetic patients show insulin resistance at the level of the synovial membrane, in addition to the systemic one, and in association with inflammatory processes, increases oxidative stress and pro-inflammatory cytokines at the joint level [21]. Thus, the synthesis of type II collagen decreases, leading to premature chondrocyte senescence and apoptosis, subchondral bone mass loss, and degenerative changes. Lipid metabolism can initiate or exacerbate the pathology by depositing lipids at the chondrocyte levels. Such pathology could increase the concentration of lipid peroxides, which generate aldehyde products that have destructive effects on the extracellular matrix and cellular components [22], and arterial hypertension causing subchondral ischemia [4].

The risk of KOA increases when associated with some MetS components [23], whereas the mortality of patients with KOA-MetS is generally higher than those without this pathology [24].

Since there are implications of MetS in increasing the risk of producing or worsening KOA, correcting its constituent elements may be very important, especially in the absence of curative treatment of degenerative knee pathology [11,23].

The present study aims to evaluate the association of KOA with MetS components represented by obesity, dyslipidemia, hyperglycemia, and the environment of origin. According to our knowledge, it is the first paper in which the environment of origin is considered a risk factor in the southeastern European region.

## 2. Materials and Methods

The clinical study included 373 outpatients admitted to the Military Emergency Clinical Hospital “Dr. Iacob Czihac” (SMUIS) Iași, Romania, based on the approval of the Ethics Committee from SMUIS issued under no. 10/A575. After thorough screening by applying inclusion/non-inclusion criteria to the 373 patients, only 85 outpatients were finally included in this study. According to the KL classification, five study groups of patients were established. The control group comprises 29 patients without KOA (grade 0 KL), and the other four KOA-affected groups are formed by 56 patients with KOA—grades I-IV KL.

The inclusion criteria were as follows: males and females with symptoms of unilateral or bilateral knee pain that correlated with clinical examination suggested the diagnosis of KOA, in association with standard standing (AP) bilateral X-rays assessed for setting the diagnosis; patients with clinically determined and recorded values of total cholesterol, LDL, HDL, triglycerides, and the glycemia in the clinical observation sheet.

The non-inclusion criteria applied were as follows: patients presenting lower limb arthroplasties; history of lower limb or pelvis fractures and previous knee surgery: ligamentoplasty, meniscectomy; inflammatory rheumatic diseases such as rheumatoid polyarthritis; other major conditions like malignant illness.

**Figure 1:**
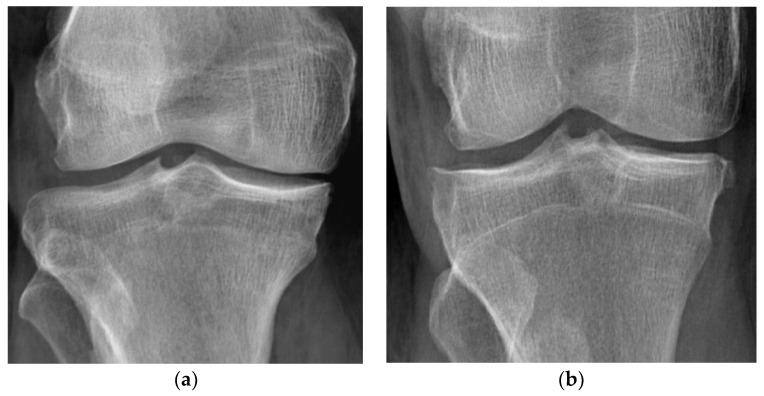

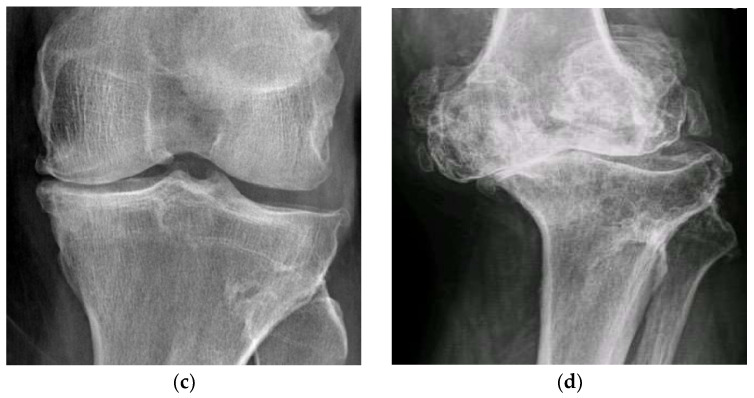
KL classification and illustration of KOA severity, SMUIS X-Ray collection of the clinical study (real cases). (**a**) KL grade I—doubtful narrowing of the joint space and possible osteophytes; (**b**) KL grade II—definite osteophytes and possible narrowing of joint space; (**c**) KL grade III—definite narrowing of the joint space, significant osteophytosis, and possible bone deformities; (**d**) KL grade IV—marked joint space narrowing accompanied by deformities, bone sclerosis, and large osteophytes.

The radiologic investigation was performed on an advanced system (Apollo DRF model from Villa Sistemi Medicali S.P.A., Buccinasco, Italy), that allowed us to record high-resolution X-ray images. The tibiofemoral joint space narrowing measurements were performed on X-ray images and subsequently digitalized using a specific software package (FCR Prima Console Viewer program). For the measurements, we used the middle portion of the lateral and medial joint spaces as a reference for each knee. Then, we determined the maximum height of the radiotransparent area between the edges of the tibiofemoral articular surfaces. The X-rays that showed a joint space of less than 5 mm were graded according to the KL scale [25], Figure 1.

The elements that constitute MetS and their values could be summarized as follows: triglycerides > 150 mg/dL, LDL > 130 mg/dL, and HDL < 40 mg/dL for masculine gender and <50 mg/dL for female patients [26], BMI ≥ 25 kg/m^2^, glycemia ≥ 110 mg/dL [27].

The ratio between weight (kg) and height square (m^2^) was applied to evaluate the BMI [28], and the patients were classified into degrees of obesity, according to the values presented in Figure S1a from Supplementary Material.

The collected blood samples were kept in laboratory conditions (at room temperature) and processed within 2 to 3 h from the collection moment.

The parameter values were obtained using two automated biochemistry analyzers equipped with turbidimetric and spectrophotometric detectors (BA400 from Biosystems, Barcelona, Spain, and ILab 650 from Instrumentation Laboratory, Barcelona, Spain), that assured a high resolution and accuracy due to the new LEDs optical system, which provides eight specific wavelengths for the spectrophotometric readings. As a reference for interpreting the obtained data, the typical values for the biological parameters were used in accordance with the results issued by an accredited laboratory [29], Figure S1b.

### Statistical Analysis

We performed an analysis of variance (ANOVA) [30] on all covariates of interest with respect to the severity of the disease. Based on the degree of the disease, we classified the patients on three levels of KOA affection, corresponding to no clinically identified condition (*n* = 29), normal KOA (*n* = 39) for KL grade I and II, and severe KOA disease (*n* = 17) for KL grade III and IV. For all covariates, the ANOVA assumptions were investigated using the Shapiro test for normality [31], as well as the Levene [32] and Fligner–Killeen [33] homogeneity of variance tests. The homogeneity of variance assumption was not violated for any of the covariates. All statistical tests were considered significant for a confidence level of 95%. A slight variation from normality was observed for glycemia, HDL, and age, indicated by the normality test and the investigation of Q-Q plots using a confidence level of 95%. All statistical tests were considered significant for a confidence level of 95%. All statistical analyses were performed in R [34]. The ggplot2 package [35] was used for all generated images, whereas the R packages like MASS [36], Hmisc [37], foreign [38], multivariance [39], tidyverse [40], caTools [11], and pROC [41] were used for ANOVA and regression analysis.

Several ordinal logistic regression models were fitted and interpreted for the considered data to assess the joint effect of the studied covariates on the disease severity. Due to the low number of unilateral diseased patients, a binomial logistic regression model could not be fitted only for those; however, we were able to assess a model for patients with bilateral disease. By determining the difference between the null deviance and the residual deviance offered by an ANOVA test of the fitted logistic regression model, we selected the three covariates that have the highest effect on the model: the environment of origin, BMI, and LDL values. We evaluated the area under the curve (AUC) for the receiver operating characteristic (ROC) curves using 85% of the data as a training set and 15% of the data as a testing set, with a split repetition of 10,000.

Although the considered variables are not entirely independent as they could interact with each other, they are expected to affect KOA incidence or severity. Our study focused on the metabolic parameters: HDL, LDL and total cholesterol, triglycerides, BMI, and glycemia. We considered them explanatory variables, whereas the disease severity was treated as the response variable, expecting that the chosen explanatory variables would explain the incidence and KOA degree evolution. As eight variables were considered, some of them can be highly correlated, meaning that their inclusion into a predictive model would result in a significant redundancy.

**Figure 2:**
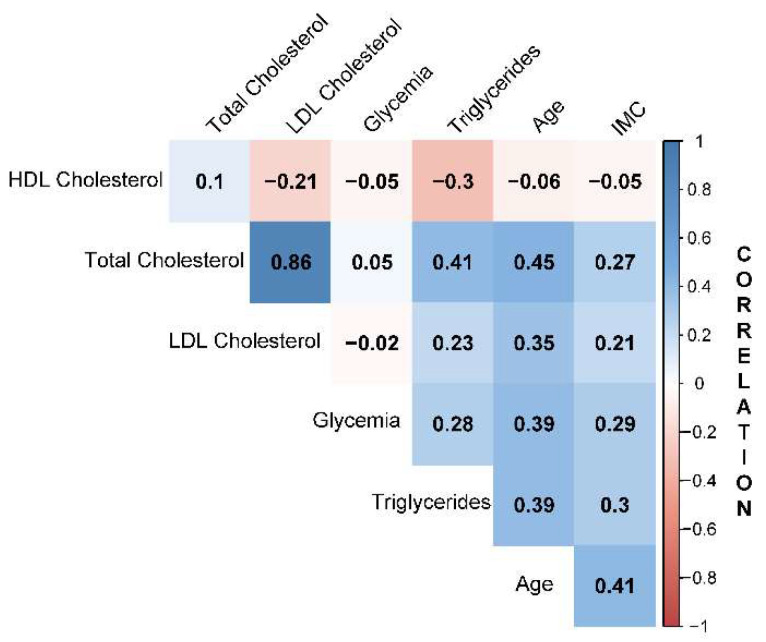
Correlation of explanatory variables.

Consequently, choosing a suitable subset of explanatory variables is necessary, as the correlations between them could generate difficulties regarding the causal interpretation. The diagram from Figure 2, presents the correlation data for the continuous explanatory variables, making it easily noticeable that each parameter’s correlation with itself equals unity. The results showed the highest correlation between the two variables, LDL, and total cholesterol. The observed negative values might suggest that HDL statistically decreases with glycemia, BMI, LDL, and triglycerides. Meanwhile, LDL decreases only with glycemia. Following this observation, a certain redundancy could be expected if both variables, LDL and total cholesterol, were included in multiple regression, so their influence on KOA incidence or severity should be balanced. Consequently, only the LDL parameter was used in the modeling as the total cholesterol parameter was highly correlated.

## 3. Results

**Figure 3:**
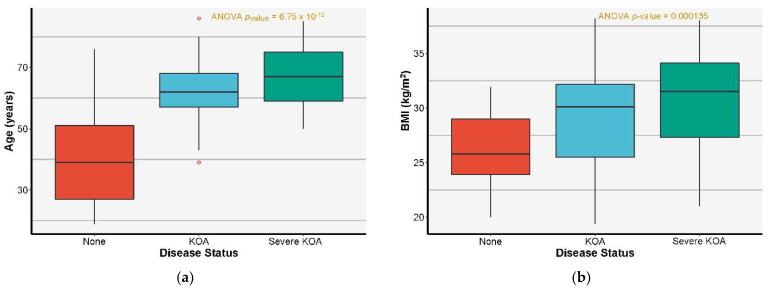
(**a**) Age distribution stratified by KOA severity with the associated ANOVA test *p*-value. (**b**) BMI distribution stratified by KOA severity with the associated ANOVA test *p*-value.

Patients between 19 and 86 years old were included in the study, with a median age of 58, out of which 34 patients were from the rural environment, whereas 51 patients were from urban areas. The ratio of 2:3 considered for the rural: urban environment ratio respects the actual rural/urban balance in Iasi County, taking into account the statistical data of the population distribution, migration of population from rural areas, immigration of population from Moldova Republic to Iasi, and the extension of the Metropolitan Area of Iasi [42]. Of the patients with a rural background, 13 were males and 21 were females, whereas from the urban environment, 29 were males and 22 were females. The total number of females included in the study was 43, whereas the number of males was 42. Figure 3a presents the patients’ age distribution, clearly indicating the known dependence between KOA affection and age. It must be noted that for patients where the prevalence of the disease increases above 50 years, the effect of the patient’s age is no longer statistically significant, having a non-significant Tukey HSD *p*-value of 0.37. Given KOA’s old age disease status, age as an explanatory variable should be considered mainly in the case of diseased patients as its weight would strongly bias any model that would include younger patients.

Out of the 56 KOA patients included in this study, 54 had tricompartimental arthritis with a genu varum deformity accusing pain mainly in the medial side of the knee; 2 patients had a valgus deformity. All patients accused function limiting knee pain and stiffness. A total of 9 patients presented unilateral KOA, whereas 47 presented bilateral KOA. The median BMI was 29 for all included patients, with a specific value of 27.15 for males and 30.50 for females. The median BMI for the rural environment was 27.45, whereas for the urban one, it was 29.40. For the rest of the analysis, the highest degree of KOA between the two knees of each patient was considered for evaluating disease severity, as is shown in Figure 3b for the BMI impact on KOA incidence and severity.

**Figure 4:**
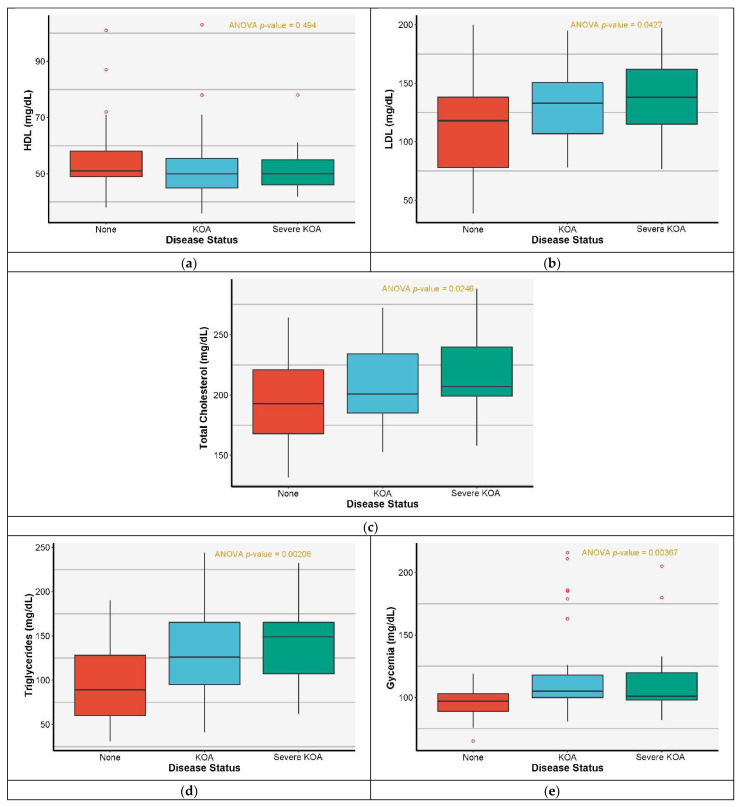
Distribution of patients’ MetS parameters values function of KOA severity with the associated ANOVA *p*-values (**a**) HDL distribution, (**b**) LDL distribution, (**c**) total cholesterol distribution, (**d**) triglycerides distribution, (**e**) glycemia distribution.

HDL had no statistically significant difference between the analyzed groups—Figure 4a, whereas the LDL and total cholesterol presented weak evidence of significance with respect to intergroup variation (ANOVA *p*-value of 0.043 and 0.025, respectively), Figure 4b.

The post hoc Tukey HSD test indicated a statistically significant difference in the total cholesterol between healthy patients (lower values, with a median of 193) and patients with severe KOA (higher values, with a median of 207) for a *p*-value of 0.026—Figure 4c.

The triglycerides showed a statistically significant difference between healthy and diseased patients with an ANOVA *p*-value of 0.002. Figure 4d highlights an important difference between healthy patients (median of 89) and KOA patients (median of 126) with a *p*-value of 0.005, pointed out by the Tukey HSD test. According to the graph from Figure 4e glycemia also exhibited a statistically significant difference with an ANOVA *p*-value of 0.003; however, in this case, we remarked the presence of outliers. As in the case of triglycerides, the most significant difference is between healthy patients (median of 97) and KOA patients (median of 105).

A number of ordinal logistic regression models were fitted to assess the joint effect of the studied covariates on the disease severity. Analyzing the Akaike information criterion [43], which allows us to evaluate what model fits the data better, we found that removing HDL and total cholesterol leads to a better model. Therefore these parameters were not included in the applied models. Moreover, the age of the patients was found during the exploratory analysis to be highly biased; the patients younger than 40 years exhibited only unilateral or no KOA diagnosis. Because of this, age was not considered to be modeled as a parameter.

Considering an ordinal logistic regression model that includes all covariates, Table 1 shows that gender, the environment of origin, BMI, and glycemia were statistically significant. As a reference, the masculine gender for sex and urban for the environment of origin were considered. The calculated parameters showed a significant decrease of 0.281 in the disease odds by having a male gender. The influence of the rural environment favored the urban one, decreasing the disease odds by 0.379, considering no variation in all the other covariates.

In comparison, a unit increase in glycemia increases the disease risk by 1.7%, whereas a unity change in BMI increases the KOA risk by 14%, keeping all the other covariates constant. However, within the frame of the applied model, the statistical *p*-value corresponding to LDL and triglycerides exceeds 0.05, signifying that such variables do not influence the KOA incidence and severity, considering a 5% significance level.

A binomial logistic regression model that could fit the data was assessed for the patients with bilateral KOA affection. Similar to the previous model, the masculine gender for sex and urban for the environment of origin were the references. Subsequently, we found that only the environment of origin is statistically significant (*p* < 0.05) (Table 2), with patients coming from rural areas (*n* = 20) having an odds ratio of 5.18, highlighting a higher disease-associated risk of developing a severe disease compared to the patients from urban areas (*n* = 27), keeping all the other covariates constant.

A mean AUC value of 0.72 was obtained, indicating acceptable discrimination [44] between a normal KOA and severe KOA disease using the corresponding fitted model with only the environment of origin as a statistically significant covariate.

**Figure 5:**
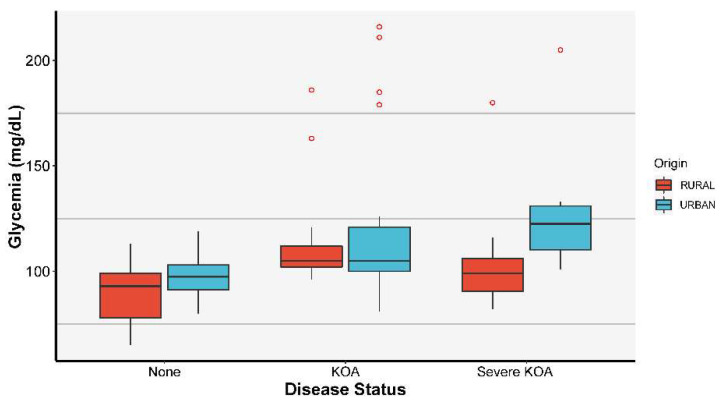
The influence of glycemia on KOA severity stratified by the environment of origin.

At the same time, we carried out statistical studies on the influence of the environment of origin in relation to the other considered parameters, highlighting the specific contribution of the environment of origin. The stratified results obtained, as shown in Figure 5 for the glycemia parameter, allowed us to follow up on the evolution of KOA severity with the considered MetS components and the environment of origin.

## 4. Discussions

Patients with KOA do not have a curative treatment, and in this context, the prevalence of the pathology is continuously increasing [1], a fact that causes the overload of medical services. Several risk factors associated with the onset or progression of KOA are represented by age, sex, or BMI [25].

KOA was initially seen as a pathology characteristic of the elderly over 65 years [19], but it has recently been observed in increasingly younger patients [45]. An essential increase in KOA prevalence is in the case of people aged 50 and over [46], especially in association with the existence of MetS [19].

Regarding sex influence, a high prevalence is found mainly in the feminine gender [47]. Twenty years ago, in patients aged 60 years, the prevalence of KOA in women was 13% and 10% in men [48]. Recent estimations show that in patients aged 65 and older, KOA occurs in 42.1% of the feminine gender, and in 31.2% of the masculine gender. In the meantime, feminine gender patients report symptoms more frequently and show significant correlations with radiological changes [3]. Nur H. et al. [49] explain these associations by the multitude of metabolic and hormonal changes that women’s bodies undergo during menopause and by the presence of MetS [50].

Our clinical study comprises patients whose average age was 58 years old, with a distribution of the total number of patients relatively homogeneous by gender, especially being a more significant number of feminine gender in rural areas (21 compared to 13 masculine gender patients).

Weight gain is one of the most important potentially modifiable risk factors [51], the consequences of obesity being represented by metabolic, biological, and immunological effects and increased mechanical stress. Thus, obesity accelerates the onset and progression of KOA, Fowler-Brown objectifying a 32% higher risk in people with a BMI increase of 5 kg/m^2^ [52]. The influence of obesity is accentuated from a young age; a high BMI at 20 years of age will increase knee pain by approximately 38% and the severity of functional limitation by 27% with advancing age [53]. Li D. et al. [54] highlight the importance of BMI for symptomatic KOA, which affected 28% of obese patients, 21.1% of overweight patients, and 15.3% of normal-weight patients. The analyses carried out on the microarchitecture of the subchondral bone highlight, in the case of obese patients, evidence of densification of the trabeculae at the level of the subchondral bone, which implies a decrease in the elastic properties and implicitly a faster cartilage degradation [55].

Following previous studies [56], we found that BMI has an essential role in the differentiation between OA patients and healthy patients (ANOVA *p*-value of 1.35 × 10^−4^), the most significant difference being observed between healthy patients with a median index of 25.80 kg/m^2^ and severe KOA with a median value of 31.50 kg/m^2^, indicated by a Tukey honest significant difference (Tukey HSD) pairwise post hoc comparison test *p*-value of 0.002. For every unit change in BMI, we remarked an increase of nearly 14% of patients affected by the KOA disease compared to the healthy ones. Gender, the environment of origin, and BMI were found to be statistically significant in the considered model that includes all covariates. According to the applied ordinal logistic regression, the female gender was observed to increase the disease odds by 3.57. Coming from a rural environment increases the odds by 2.63, considering no variation in all the other covariates.

Regarding the presence of diabetes mellitus (DM) in the context of KOA, some more severe changes from the imaging point of view were highlighted, especially in the male gender [57]. In the meantime, the conclusions of Williams et al. argue that the onset or progression of KOA is influenced by high glycemia levels [58].

In addition, the risk analysis of the need for arthroplasty interventions in patients with KOA revealed that DM is not a risk factor in this sense but rather obesity [59]. Al-Jarallah K. et al. [60] report that diabetic patients treated with insulin had lower severity of radiologically detectable osteophytosis than people with diabetes who did not receive insulin. Although its mode of action in KOA is not yet known, extensive studies are needed to investigate the mechanisms and influence that intraarticular insulin administration might have a benefit [60]. Other researchers associate antidiabetic treatment with a slowdown in the progression of KOA but without a decrease in the pathology incidence, suggesting a potential anti-inflammatory and protective effect at the joint level [61].

The results of our study reveal that elevated glycemia values have a statistically significant influence on the diagnosis of KOA, with the patients having an average value of 105 mg/dL, compared to the control group, which had a value of 97 mg/dL. Glycemia is statistically significant in the ordinal logistic regression model that includes all covariates. A unit increase in glycemia increases the disease risk by 1.7%, keeping all the other covariates constant. An explanation of how DM intervenes in the KOA was given by Laiguillon M.C. [62], who claims that DM produces an increased response of the articular cartilage to certain types of interleukins mediated by oxidative stress and increased inflammation in the joints [22,62,63].

The link between DM and KOA can also be explained by the causal role that glycation end-products possibly have in generating the pathology. Hydroximidazolone derived from methylglyoxal, one of the main glycation end-products, is found in a higher concentration in the synovial fluid of patients with DM associated with KOA [64]. Another study suggests that DM may influence both the progression and severity of KOA and the therapeutic outcomes of specific treatments. Still, more research is needed to evaluate these hypotheses [65]. The presence of MetS has been correlated with more severe KOA symptomatology [56], and a family history of hypercholesterolemia is considered to be a risk factor for knee pain, a one unit increase in total cholesterol, or LDL fraction resulting in an 8% and 6% increased risk of pain, respectively [66]. At the same time, the evaluation of the influence of triglyceride values highlights the fact that an increase of one unit causes a 5% higher risk for patients to present knee pain and a 9% higher risk for them to present clinically objective changes, compared to people with typical values of triglycerides [66].

In the present study, triglyceride values showed significant mean differences between the control group (89 mg/dL) and the test group (126 mg/dL). The importance of addressing KOA in the context of the phenotype associated with MetS is supported by the evidence of an increased risk of pathology in dyslipidemia, thus suggesting that such imbalances could be risk factors, presenting positive associations in most of the technical articles [67].

There were reported average values of 245.0 ± 25.1 mg/dL in patients with KOA, respectively, 233.0 ± 17.5 mg/dL for people without KOA in the case of total cholesterol, and LDL values of 126.5 ± 20.7 mg/dL for the group without KOA and 136.9 ± 15.9 mg/dL for people with KOA [67]. In our research, total cholesterol and LDL showed statistically significant influences in intergroup variations, with an important significance of total cholesterol, which presented an average of 193.0 mg/dL in the control group, respectively 207.0 mg/dL in the test group.

Additionally, some analyses performed on laboratory animals reveal a higher risk of KOA in the low HDL and high LDL cases; such unbalanced HDL-LDL values potentiate inflammation at the synovial level and the formation of osteophytes [68], as well as injuries at the bone morrow level [69]. Consequently, specific treatments for cholesterol value adjustments were applied, expecting a good answer as a therapy for KOA. Depending on the type and duration of statin administration, it has been shown to have different effects on the perception of knee pain. Thus, administration over four years did not cause a change in pain intensity. Still, over five years, patients who used atorvastatin declared that the pain did not intensify. On the other hand, people who were treated with rosuvastatin reported more severe knee pain. Therefore, the authors considered that some statins might also have anti-inflammatory or protective effects, which should be studied more carefully [70,71,72]. In general, the findings of the evaluations so far reveal dyslipidemia’s involvement to varying degrees in the arthrosis pathogenesis, which requires further research on the beneficial effects of antihyperlipidemic therapies on KOA [73].

The present study proved that regardless of the mathematical model applied for the statistical processing of patients’ clinical data, the origin environment was statistically significant. Thus, considering all patients from the target group of the clinical study, the rural environment increases the odds by 2.63 if all other covariates are kept constant. When considering only the patients with bilateral disease by applying a binomial logistic regression model, it was assessed that only the environment of origin is statistically significant. Although the number of patients from rural areas is lower (*n* = 20) compared to those from urban areas (*n* = 27), a 5.18 times higher risk of developing a severe disease for rural people was determined compared to urban patients. The calculated statistical parameters highlighted acceptable discrimination between a grade I or II KOA and severe KOA disease when applying the fitted model with only the environment of origin as a statistically significant covariate.

Regarding the influence of the patients’ environment of origin, in addition to the critical parameters discussed here, psychological factors and living conditions, often dependent on the geographical area studied, could impact the evolution of KOA. Very recently, for the geographical area of Thailand, A. Siripongpan and B. Sindhupakorn [74] showed correlations similar to our study between patients living in urban and rural areas. The authors linked these differences with the quality of life represented by physical, mental, social, and environmental health. Marisa Coetzee et al. [75] highlighted the geographical area of the Western Cape, South Africa, that deficient education in the rural environment could determine the aggravating factors of KOA in the approach to medical services and the inefficient delivery of health services. Our results are in accordance with previous studies proving that the KOA prevalence is higher in rural environments [76,77]. The present article refers to a group of patients studied in the southeastern European region, but the observations of the previous studies cited here highlight that the typology of the KOA pathology presents complex correlations with the environment of origin, being an ever-current research topic that must be addressed in a multidisciplinary and dynamic manner, depending on the particular evolution of the society of the studied environment.

Like many clinical trials, the present study has both strengths and limitations. First of all, the statistical research was carried out taking into account an important number of factors specific to the MetS: HDL, LDL and total cholesterol, triglycerides, BMI, and blood sugar assessed the interactions between the considered variables and adjusted through mathematical modeling the factors that can generate redundant results, such as LDL and total cholesterol, thus improving the outcomes. Then, it is the first study from the southeastern European region that relates the environment of origin to the KOA incidence and severity performed on a balanced sample of the population from Iasi County in Romania, showing that the risk of developing a severe KOA is over five times higher for rural people compared to urban patients. However, we must acknowledge the study’s limitations, as the applied design precludes the influence of HTA, work and social conditions, or sports habits. Moreover, due to objective constraints, primarily related to the coronavirus (COVID-19) pandemic, the number of subjects included in the study might be considered somewhat limited. Because the patients included in the study were outpatients, certain important indices such as blood pressure, correlation with sports activities, or occupational history were not included. On the other hand, we consider that blood pressure can be a variable parameter for which patients generally already have impaired treatment. In addition, the multifactorial etiology of KOA necessitates, in general, discussing this subject in terms of a limited number of factors. In our opinion, the other factors mentioned above that were not taken into account here do not result in errors in the conclusions. However, their inclusion would have undoubtedly aided in a better understanding of KOA prevalence. As presented in this section, several findings from this study have already been incorporated into the current consensus about age, blood sugar, and BMI’s impact on KOA, which serves as a validation milestone of the present study. Nevertheless, as no other investigation approached the association between the environment of origin and KOA presence for a southeastern European population segment, the significance of the present study should not be alleviated due to the limited number of participants.

## 5. Conclusions

Our study supports the specialized international literature that emphasizes the connection between elements of MetS and KOA, also particularizing the importance of the environment of origin, patients from rural areas being more affected by disease compared to those from urban areas. In this sense, it would be interesting to approach the problem from the perspective of occupations specific to the environment of origin in correlation with the other risk factors characteristic of geographical regions, which may also include climatic conditions.

The present study revealed that glycemia values have a statistically significant influence on the diagnosis of KOA. Thus a unit increase in glycemia increases the disease risk by 1.7%. Meanwhile, every unit change in BMI generated about a 14% increase in patients affected by the KOA disease compared to the healthy ones. In addition, the considered parameters highlighted a significant decrease of 0.281 in the disease odds by having a male gender.

Our study showed that the patients’ environment of origin was statistically significant. Considering only patients with bilateral disease by applying a binomial logistic regression model, only the environment of origin was determined to be statistically significant. Thus, considering all patients in the target group of the clinical trial, rurality increases the odds of having KOA of various degrees of severity by 2.63.

The presented clinical study, according to our knowledge, is the first one that takes into account as a risk factor the environment of origin of the participating subjects from the southeastern European region. Thus, the metabolic phenotype of KOA may be plausible and suggest individualized treatment with an important focus on risk factors for an expected better clinical response from patients.

## Figures and Tables

**Table 1:** An ordinal logistic regression model including all considered covariates.

	Odds Ratio (OR)	Confidence Interval 2.5%	Confidence Interval 97.5%	*p*-Value
Sex (M)	0.281	0.103	0.727	0.010
The environment of origin (URBAN)	0.379	0.144	0.960	0.044
BMI	1.140	1.018	1.284	0.026
LDL	1.012	0.998	1.027	0.095
Triglyceride	1.008	0.998	1.018	0.132
Glycemia	1.017	1.001	1.035	0.042

**Table 2:** Binomial logistic regression model for bilateral KOA affection.

	Odds Ratio (OR)	Confidence Interval 2.5%	Confidence Interval 97.5%	*p*-Value
Sex (M)	1.212	0.272	5.431	0.797
The environment of origin (URBAN)	0.194	0.043	0.743	0.022
BMI	1.076	0.920	1.277	0.370
LDL	1.011	0.987	1.037	0.386
Triglyceride	0.997	0.983	1.011	0.720
Glycemia	1.002	0.979	1.023	0.888

## Data Availability

Not applicable.

## Supplementary Materials

The following supporting information can be downloaded at: https://www.mdpi.com/article/10.3390/jcm11247302/s1. Figure S1: (**a**) BMI variation determining the degrees of obesity; (**b**) Values of the metabolic parameters within the normal range.

## Abbreviations

ANOVA	analysis of variance
AUC	area under the curve
BMI	body mass index
DM	diabetes mellitus
HDL	high-density lipoprotein
KL	Kellgren–Lawrence classification
KOA	knee osteoarthritis
LDL	low-density lipoprotein
MetS	metabolic syndrome
ROC	receiver operating characteristic curves
SMUIS	Military Emergency Clinical Hospital “Dr. Iacob Czihac” (SMUIS) Iași, Romania
