# Supplementary material for: Knee Osteoarthritis in Relation to the Risk Factors of the Metabolic Syndrome Components and Environment of Origin
Source: J Clin Med. 2022 Dec 8;11(24):7302. doi: 10.3390/jcm11247302 (PMC9781325; doi:10.3390/jcm11247302)
Supplement: Supplementary file 1 [file jcm-11-07302-s001.zip › jcm-2012805-supplementary.pdf]

Journal of
Clinical Medicine

Supplementary Materials

Knee Osteoarthritis in Relation to the Risk Factors of the Metabolic Syndrome Components and Environment of Origin

Nicoleta Bianca Tudorachi [1], Tiberiu Totu [2], Iuliana Eva [3], Bogdan Bărbieru [4], Eugenia Eftimie Totu [5,*], Adrian Fifere [6,*], Tudor Pinteală [7], Paul-Dan Sîrbu [7] and Valeriu Ardeleanu [1]

[1] Faculty of Medicine, "Ovidius" University of Constanța, Mamaia Boulevard 124, 900527 Constanța, Romania
[2] Department of Health Sciences and Technology (D-HEST), ETH Zurich, 8093 Zurich, Switzerland
[3] Radiology and Medical Imaging Laboratory, "Iacob Czihac" Emergency Military Clinical Hospital, 7-9 General Henri Mathias Berthelot St., 700483 Iași, Romania
[4] Department of Orthopedics and Traumatology, "Iacob Czihac" Emergency Military Clinical Hospital, 7-9 General Henri Mathias Berthelot St., 700483 Iași, Romania
[5] Department of Analytical Chemistry and Environmental Engineering, Faculty of Industrial Chemistry and Biotechnologies, University Politehnica of Bucharest, 1-5 Polizu Street, Sector 1, 011061 Bucharest, Romania
[6] Centre of Advanced Research in Bionanoconjugates and Biopolymers, "Petru Poni" Institute of Macromolecular Chemistry, 41A Grigore Ghica Voda Alley, 700487 Iași, Romania
[7] *Department* of *Orthopedics* and *Traumatology*, Faculty of Medicine, Grigore T Popa University of Medicine and Pharmacy, 16 University Street, 7001 Iași, Romania
* Correspondence: eugenia.totu@upb.ro (E.E.T.); fifere@icmpp.ro (A.F.)

The body mas index (BMI) was assesses as the ratio between weight (kg) and height square (m^2), the patients being included in different categories obesity, according to the values presented in Figure S1.a.

The collected blood samples from the patients recorded for the clinical study were used to determine the biological parameters of interest. The typical values for the biological parameters are presented in Figure S2.b.

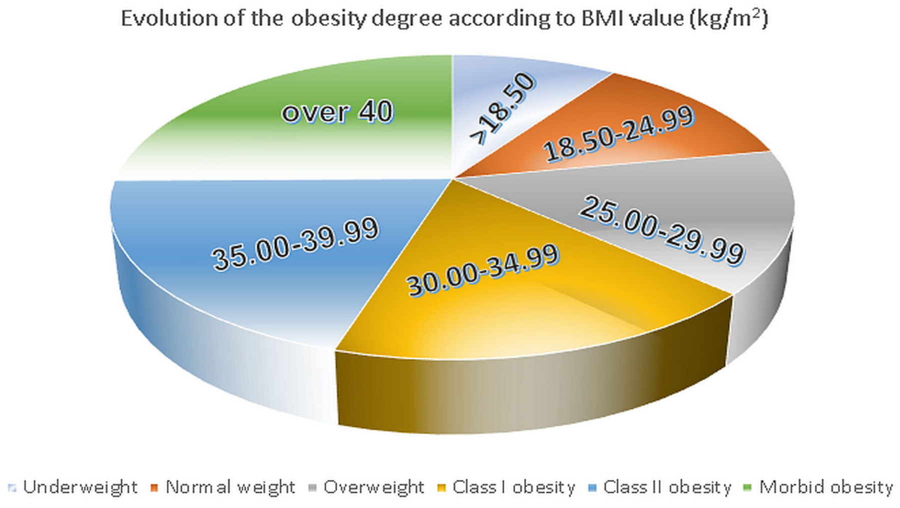

(**a**)

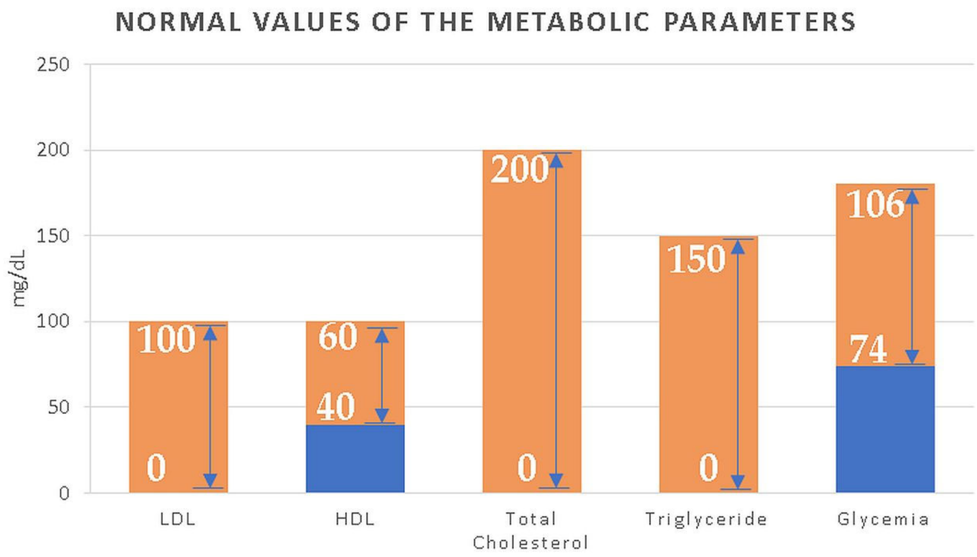

(**b**)

Figure S1. (**a**) BMI variation determining the degrees of obesity; (**b**)Values of the metabolic parameters within the normal range.
